# Effect of Tropicamide on crystalline Lens rise in low-to-moderate myopic eyes

**DOI:** 10.1186/s12886-020-01594-8

**Published:** 2020-08-10

**Authors:** Zhuoyi Chen, Tao Li, Meiyan Li, Ye Xu, Xingtao Zhou

**Affiliations:** 1 Eye Institute and Department of Ophthalmology, Eye & ENT Hospital, Fudan University, Shanghai, China; 2 NHC Key Laboratory of Myopia (Fudan University); Key Laboratory of Myopia, Chinese Academy of Medical Sciences, Shanghai, China; 3 Shanghai Research Center of Ophthalmology and Optometry, Shanghai, China

**Keywords:** Tropicamide, Crystalline Lens rise, Myopia

## Abstract

**Background:**

Cycloplegics have been reported to induce changes in the lens thickness. However, the studies of correlation between cycloplegia and the lens position are limited. This study aims to investigate changes in crystalline lens rise (CLR) and other anterior segment parameters after inducing cycloplegia with tropicamide.

**Methods:**

In this consecutive case study, 39 children (20 boys and 19 girls; mean age, 9.51 ± 1.75 years, mean spherical equivalence [SE], − 1.9 ± 1.5 D) with low-to moderate myopia were examined using CASIA 2 both before and after 30 min of administering 5-cycles (each 5 min apart) of 0.5% tropicamide. Measurements included CLR, crystalline lens thickness (CLT), mean radius of curvature of the anterior/posterior surface of the lens (Rf_ave/Rb_ave), anterior chamber depth (ACD), anterior chamber width (ACW), and central corneal thickness (CCT). Correlations of CLT and CLR with ACD, SE, and age were assessed respectively.

**Results:**

CLT and CLR decreased significantly after cycloplegia (*p* < 0.001 and *p* < 0.001, respectively); whereas CCT, ACD, and Rf_ave increased (*p* = 0.008, *p* < 0.001, *p* < 0.001, respectively). A positive correlation was found between CLR and SE (*r* = 0.565, *p* < 0.001). However, a negative correlation between ACD and CLR was found before and after cycloplegia (*r* = − 0.430, *p* = 0.006; *r* = − 0.342, *p* = 0.035, respectively).

**Conclusions:**

The crystalline lens appeared thinner and moved backward after cycloplegia. ACD increased mainly due to the backward movement of the crystalline lens. These results aid in elucidating the impact of crystalline lens changes during the process of accommodation.

## Background

The global prevalence of myopia increased to 28.3% in 2010 and is estimated to be 49.8% by 2050 with this trend [1]. This prevalence is rising in children as well, making myopia an important health concern for children worldwide [2–5]. Evidence has shown that anterior chamber changes occur along with axial length growth in myopic children’s eyes [6–8]. But cycloplegics, agents for paralyzing the ciliary muscle, have been reported to induce changes in the anterior segment and crystalline lens to overcome the myopic shift [9–11].

Cycloplegic agents, namely tropicamide, cyclopentolate, and atropine, are generally applied in ocular examinations and for myopia control [12–14]. Among these, tropicamide is widely used owing to its short duration of action and less side effects. Previous reports have investigated changes in the anterior chamber depth (ACD) and central corneal thickness (CCT) after cycloplegia [15–19]. However, the study of the correlation between cycloplegia and the crystalline lens rise (CLR), the distance between the anterior surface of the crystalline lens and the line of angle recess, had been very difficult until CASIA2 (CASIA2, TOMEY, Nagoya, Japan) was introduced.

CASIA2 is an anterior segment optical coherence tomography (AS-OCT) which can scan the anterior segment at a depth of 13 mm. Therefore, it can offer an opportunity to investigate the changes in the crystalline lens and anterior segment more comprehensively and aid to understand eye biometrics in myopic children better. According to our knowledge, ours is the first study to observe changes in CLR using CASIA2 after inducing cycloplegia.

## Methods

### Patients

This prospective, consecutive case study was approved by the Ethics Committee of Shanghai EENT Hospital and was performed in accordance with the Declaration of Helsinki (Approval Number: 2016037) in November 2018. Children (age, < 15 years) with low to moderate myopia were eligible for this study. Sample size was determined as 20 via the following formula: $n=\frac{{\left({Z}_{\alpha /2}+{Z}_{\beta}\right)}^2\ast {\sigma}^2}{\delta^2}$, where the power was set to 90% and the alpha error was 0.05. Exclusion criteria were as follows: active eye pathology or systemic diseases with eye involvement, history of eye surgery, uveitis, glaucoma, and cataract and incomplete examinations due to lack of cooperation. A signed informed consent has been obtained from each patient before the study.

### Examination procedure

AS-OCT examinations were performed using CASIA2, (TOMEY, Nagoya, Japan), an automatic and non-invasive device. Patients were asked to take a proper position and fixed on the red target inside the machine. The operator aligned the corneal apex and the distance until a blue cross appeared on the image. Then, patients were instructed to blink twice and open the eyes wider. After pressing the Capture button, the instrument automatically produced 16 tomographic images from 32 directions for lens biometry.

Post-cycloplegia examination was performed 30 min after administering 5 cycles (each cycle 5 min apart) of 0.5% tropicamide, which is in agreement with previous research [11]. To maintain accuracy, all examinations were done three times by the same ophthalmologist. Measurements included CLR, crystalline lens thickness (CLT), mean radius of curvature of the anterior/posterior surface of the lens (Rf_ave/ Rb_ave), ACD, anterior chamber width (ACW), and CCT. The results of manifest refraction would be used to calculate the spherical equivalent (SE).

### Statistical analysis

All statistical analyses were performed by SPSS software (Version.25.0; IBM, Armonk, NY, USA). Descriptive statistics are expressed as mean ± SD. Shapiro-Wilk test was used to test the normal distribution. All measurements obtained before and after cycloplegia induction were compared using the paired t test or Wilcoxon test. Pearson’s linear correlation was used to determine the relationship between ACD and CLR, post-cycloplegia SE and among post-cycloplegia CLT and CLR. A *p* value of less than .05 was considered statistically significant.

## Results

This study included 39 eyes of 39 patients, among which 20 (51.3%) were male and 19 (48.7%) were female. The mean age was 9.51 ± 1.75 years old (range, 6–13 years). The SE ranged from − 0.25 D to − 5.75 D, and the mean SE was − 1.9 ± 1.5 D.

**Fig. 1:**
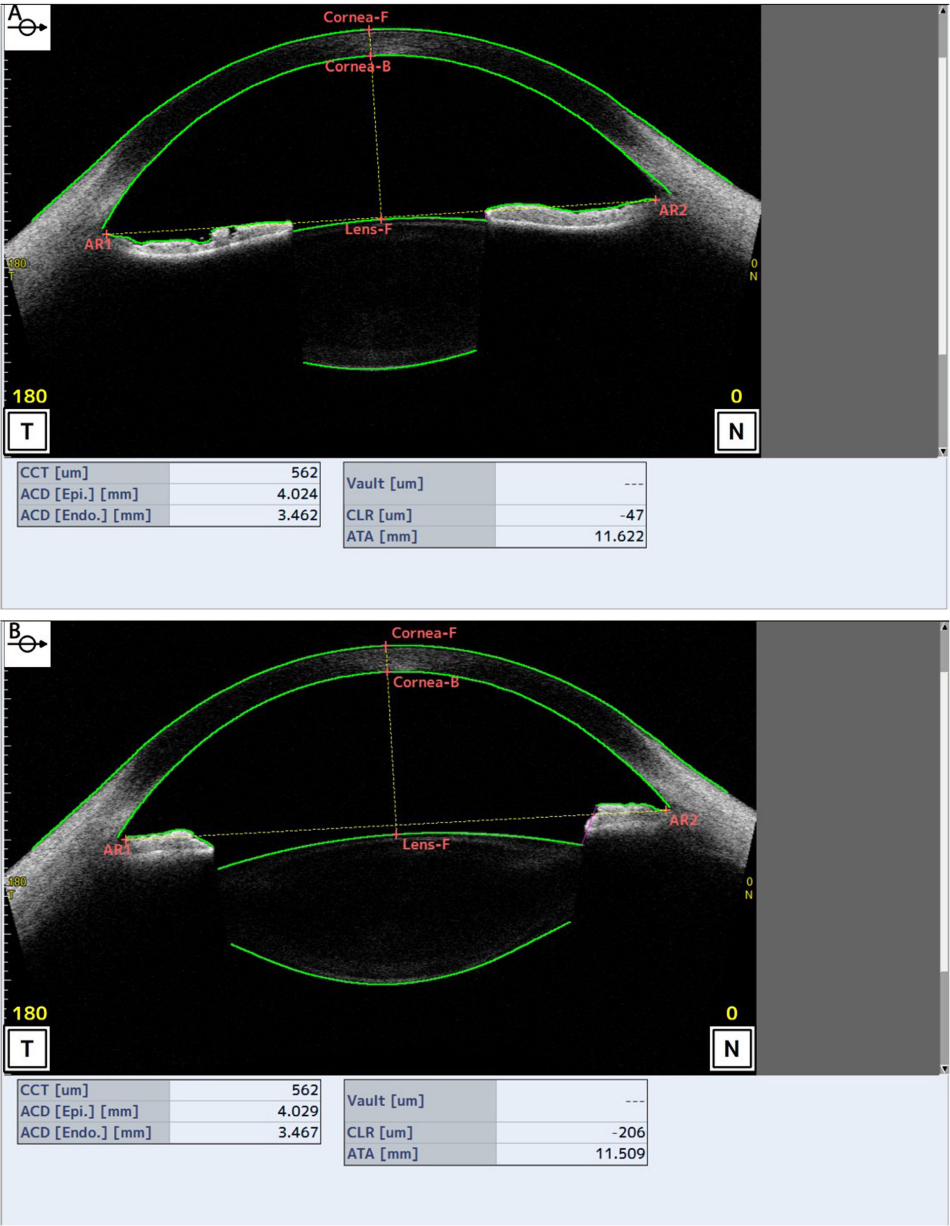
Example images of anterior segment and change of CLR at 180-degree axis of the same patient captured by CASIA2(**a**: before cycloplegia; **b**: after cycloplegia; AR: angle recess)

**Fig. 2:**
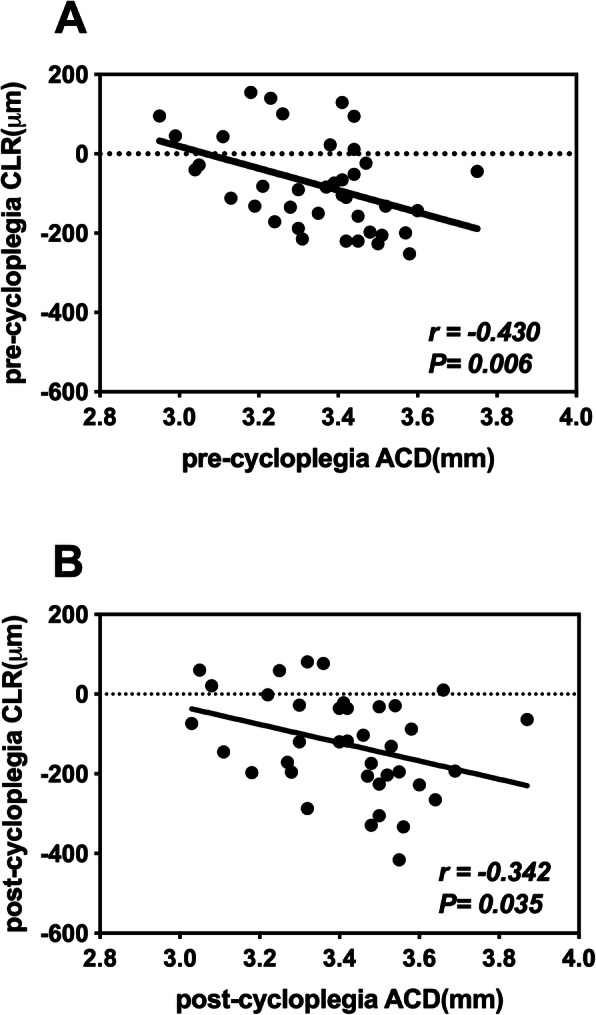
Relationship between ACD (mm) and CLR (μm) before and after cycloplegia (**a**: before; **b**: after)

All measurements before and after tropicamide application are listed in Table 1. After cycloplegia, CCT, ACD, and Rf_ave increased 1.44 ± 3.20 μm (*p* = 0.008), 0.07 ± 0.05 mm (*p* < 0.001) and 1.00 ± 0.40 μm (*p* < 0.001), respectively. However, post-cycloplegia CLT and CLR decreased and the decrements were − 0.03 ± 0.03 mm (*p* < 0.001) and − 58.55 ± 82.92 μm (*p* < 0.001), respectively (Fig. 1). No significant change was noted in the measurements of ACW and Rf_ave (*p* = 0.122 and *p* = 0.169). A negative correlation was found between ACD and CLR both before and after cycloplegia (*r* = − 0.430, *p* = 0.006 and *r* = − 0.342, *p* = 0.035, respectively; Fig. 2).

**Table 1:** Changes of Ocular Measurements Before and After Tropicamide Cycloplegia

	Before cycloplegia Mean ± SD	After cycloplegia Mean ± SD	Diff	SD	95%CI		p
					Lower	Upper	
CCT, μm	545.92 ± 29.84	547.36 ± 30.36	1.44	3.20	0.40	2.47	0.008^a*^
ACD, mm	3.35 ± 0.18	3.41 ± 0.18	0.07	0.05	0.05	0.08	< 0.001^a*^
ACW, mm	11.77 ± 0.39	11.73 ± 0.39	−0.05	0.17	−0.10	0.01	0.122^b^
CLT, mm	3.37 ± 0.15	3.34 ± 0.14	−0.03	0.03	−0.04	− 0.03	< 0.001^a*^
CLR, μm	−77.27 ± 114.57	−135.82 ± 139.16	−58.55	82.92	−85.43	−31.67	< 0.001^a*^
Rf_ave, mm	12.70 ± 1.26	13.70 ± 1.21	1.00	0.40	0.86	1.15	< 0.001^a*^
Rb_ave, mm	−5.98 ± 0.36	−5.90 ± 0.43	0.08	0.34	0.19	−0.03	0.169^a^

Data is expressed as mean ± SD

*CCT* Central cornea thickness, *ACD* Anterior chamber depth, *ACW* Anterior chamber width, *CLT* Crystalline lens thickness, *CLR* Crystalline lens rise, *Rf* Radius of lens posterior surface, *Rb* Radius of lens posterior surface, *ave*. Average, *SD* Standard deviation, *CI* Confidence interval

^*^ significant differences(*p < .05)

^a^ Paired t test. ^b^ Wilcoxon test

### Correlations between post-cycloplegia CLT, CLR, and SE

**Fig. 3:**
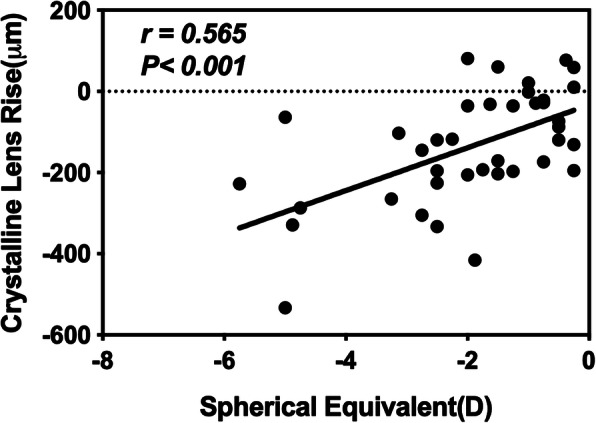
Relationship between CLR (μm) and SE(D) after cycloplegia

Pearson’s linear regression was used to determine the possible relationship. A positive correlation was found between CLR and SE (*r* = 0.565, *p* < 0.001; Fig. 3), whereas no correlation was found between CLT and SE (*r* = 0.124, *p* = 0.453). In addition, no relationship was found among CLT, CLR and age.

## Discussion

How anterior segment and crystalline lens change after cycloplegia in low to moderate myopic children is well worth discussing. In addition to CLR, this work also investigated into several other anterior segment parameters including ACD, ACW, and mean radius of curvature of the anterior/posterior surface of the lens.

ACD increased significantly (*p* < 0.001) after cycloplegia in the current study. The increase in ACD was 0.07 mm, which lies in the established range of 0.18 mm to 0.06 mm [11, 16, 20, 21]. Additionally, a negative correlation was found between ACD and CLR both before and after cycloplegia (*r* = − 0.433, *p* = 0.006 and *r* = − 0.339, *p* = 0.038, respectively), indicating an association between ACD and CLR. According to the results of this study, a 58.55 μm decrease in CLR is mostly due to the change in lens position, and only attributes 0.015 mm (1/2 change in CLT) to lens thinning. Therefore, the increase in ACD would be mainly due to the backward movement of the crystalline lens. ACW was considered to be stable after cycloplegia. To our knowledge, few studies have described changes in ACW with accommodation. Du’s study [22] demonstrated that ACW did not change during maximal accommodation in healthy eyes. Chen also found no significant change in ACW in intraocular lenses eyes [23]. These results are in agreement with ours. However, the present study focuses only on the width from nasal to temporal quadrants, whereas changes in vertical ACW still needs further investigation.

The morphology changes in the lens after cycloplegia, including thinning of thickness, backward movement, and flattening of the anterior surface, were observed significantly in our study. These changes occur due to ciliary muscle relaxation and are in agreement with the most widely accepted accommodation theory proposed by Helmholz [24]. CLR, the distance between lens anterior surface and the line of angle recess, is an important parameter representing the lens positon on the vertical line. The mean (±SD) of post-cycloplegia CLR was − 135.82 ± 139.1 μm. Here, the large SD may indicate large differences in CLR at individual level. A few previous studies have been conducted on changes in lens in myopic children and few have mentioned lens position or CLR [11, 25]. The decrease in CLR with accommodation is in agreement with results reported by Yan et al. [25] and Baikoff et al. [26] Furthermore, CLR was found to have a positive correlation with SE (r = 0.537, *p* = 0.001), which indicated a deeper lens position in a more myopic eye. One explanation to this is that a deep lens position before myopia onset can result in hyperopic defocus, contributing to myopia progression. Experiments have demonstrated that negative lenses induce myopia through hyperopic defocus in chicken and pig models [27, 28]. Another explanation is that the crystalline lens may move backwards gradually with myopia progression. Several long-term observations on myopic children have described the increase in ACD [6–8]. Since axial length growth is commonly observed in myopic progression [29, 30], is there an opportunity for a crystalline lens to move backwards with adaptation to axial length growth? This question is well worth investigating in the future.

Rf_ave, which has been mentioned before, showed that the anterior surface of the crystalline lens became flatter after cycloplegia. Although Rb_ave value showed no significant change (*p* = 0.169), the 0.08-mm mean shift in Rb_ave indicated that the posterior surface was steeper after cycloplegia on average. In Pablo’s investigation on accommodation, anterior and posterior surface changed at rates of 0.78 ± 0.18 mm and 0.13 ± 0.07 mm per D, respectively [31]. In addition, Sun also observed a steeper change in anterior surface compared with the posterior surface with accommodation [32]. These literatures indicated that the change in refractive power of the crystalline lens was mainly attributed to the anterior surface. Here in this study, the refractive error difference was considerably smaller compared with the vergence performed in above studies on accommodation; hence, posterior surface change should be minor and more difficult to be observed. In this case, the change in Rb_ave after tropicamide cycloplegia still warrants further precise measurement.

Our study has limitations. Firstly, this was a cross-sectional observation study and further long-term studies are required to prove our data. Secondly, the sample size of this study was small. Thirdly, these study did not include a control group of normal or high myopic children. Hence, our findings may be limited to low-to-moderate myopia.

## Conclusions

The crystalline lens appears thinner and moves backward after cycloplegia induction. Increase in ACD is mainly due to the backward movement of the crystalline lens. Our attempt to study these parameters with CASIA2 is new and advantageous for elucidating changes in crystalline lens with accommodation. In addition, these results are useful for physicians to read pediatric eye biometrics.

## Data Availability

Data and materials are available upon request from the corresponding author.

## Abbreviations

CLR: Crystalline lens rise
CLT: Crystalline lens thickness
Rf_ave: Rb_ave Mean radius of curvature of the anterior/posterior surface of the lens
ACD: Anterior chamber depth
ACW: Anterior chamber width
CCT: Central corneal thickness
SE: Spherical equivalent
